# Alkaline Phosphatase from Venom of the Endoparasitoid Wasp, *Pteromalus puparum*

**DOI:** 10.1673/031.010.1401

**Published:** 2010-03-04

**Authors:** Jia-Ying Zhu, Gong Yin Ye, Qi Fang, Cui Hu

**Affiliations:** ^1^ State Key Laboratory of Rice Biology & Key laboratory of Molecular Biology of Crop Pathology and Insects of Ministry of Agriculture, Institute of Insect Sciences, Zhejiang University, Hangzhou 310029, China; ^2^ Key Laboratory of Forest Disaster Warning and Control of Yunnan Province & Key laboratory of Southwest Mountain Forest Resources Conservation and Utilization of Ministry of Education, Southwest Forestry University, Kunming 650224, China

**Keywords:** histochemistry, cloning

## Abstract

Using chromogenic substrates 5-bromo-4-chloro-3′-indolyl phosphate and nitro blue tetrazolium, alkaline phosphatase (ALPase) was histochemically detected in the venom apparatus of an endoparasitoid wasp, *Pteromalus puparum* L. (Hymenoptera: Pteromalidae). Ultrastructural observations demonstrated its presence in the secretory vesicles and nuclei of the venom gland secretory cells. Using p-nitrophenyl phosphate as substrate to measure enzyme activity, the venom ALPase was found to be temperature dependent with bivalent cation effects. The full-length cDNA sequence of ALPase was amplified from the cDNA library of the venom apparatus of *P. puparum*, providing the first molecular characterization of ALPase in the venom of a parasitoid wasp. The cDNA consisted of 2645 bp with a 1623 bp open reading frame coding for 541 deduced amino acids with a predicted molecular mass of 59.83 kDa and pI of 6.98. Using multiple sequence alignment, the deduced amino acid sequence shared high identity to its counterparts from other insects. A signal peptide and a long conserved ALPase gene family signature sequence were observed. The amino acid sequence of this venom protein was characterized with different potential glycosylation, myristoylation, phosphorylation sites and metal ligand sites. The transcript of the ALPase gene was detected by RT-PCR in the venom apparatus with development related expression after adult wasp emergence, suggesting a possible correlation with the oviposition process.

## Introduction

Parasitoids spend part of their life as endo or ectoparasites of other invertebrates. During this process, their eggs and larvae contend with the immune defense responses of the hosts. Several mechanisms have evolved that enable parasitoids to escape or suppress host immune responses, which vary depending on the parasitoid species (Beckage and Gelman 2004; Renault et al. 2005; Pennacchio and Strand 2006). These include co-injection of polydnavirus (PDVs) or maternal factors, such as venom or ovarian protein with their eggs at the time of oviposition (Lanzrein et al. 1998; Beckage and Gelman 2004; Espagne et al. 2004; Moreau and Guillot 2005; Asgari 2006). These factors have often been shown to mediate suppression of the host immuneresponse. However, in certain parasitoid species, venom plays a major role in ensuring the survival and development of the parasitoid progeny in vivo (Dani et al. 2005). Despite an increasing number of peptides, proteins, and enzymes reported from the venoms of ichneumonid, braconid or eucoilid wasps (Moreau and Guillot 2005), understanding venom composition remains fragmental.

Parasitoid venoms seem to be rich in enzymes, and these enzymes vary among different parasitoid species that have highly specialized adaptations to their host range (Whitfield 1998; Schmidt et al. 2001; Dani et al. 2005). About thirty of them have been identified by enzymatic assays, immunostaining or molecular sequence. Notably, some of them have been reported to possess interesting physiological functions involving in destroying the host's immune system. For example, a serine proteinase homolog of *Cotesia rubecula* venom can interfere with the prophenoloxidase activation cascade, which inhibits melanization of host hemolymph (Asgari et al. 2003); phenoloxidase of *Nasonia vitripennis* venom is critical in the pathway leading to cell death (Abt and Rivers 2007); and γ-glutamyl transpeptidase of *Aphidius ervi* venom can induce apoptosis in host ovarioles by generating an alteration of glutathione metabolism and consequent oxidative stress (Falabella et al. 2007). The detailed exploration of diverse enzymes of parasitoid venoms is useful to contribute to the definition of the molecular bases of host-parasitoid interactions. Nevertheless, just few of them have been revealed. Moreover, among the available enzymes, there has been little information on the study of their exact biochemical and molecular natures.

Alkaline phosphatase (ALPase) (E.C. 3.1.3.1) has been characterized as an important component of venoms of some snakes and spiders (Rodrigues et al. 2006). In insects, several ALPases have been characterized in the brains of *Bombyx mori, Drosophila melanogaster, Ceratitis capitata, Culex tarsalis, Schistocerca americana* and *Bemisia tabaci*, as well as the Malpighian tubules, salivary glands, saliva, etc (Houk and Hardy 1984; Bourtzis et al. 1993; Chang et al. 1993; Itoh et al. 1999; Yang et al. 2000; Funk 2001), but there have been only few reports of ALPase from insect venoms. In these cases, ALPase was detected in ant venoms of *Paraponera cribrinodis* and *Pogonomyrmex badius* (Schmidt et al. 1986) and this enzyme was also reported to be present in honeybee venom (Hoffman 1977). In parasitoid wasps, ALPase has been reported in venom of the pupal endoparasitoid, *Pimpla hypochondriaca*, using the API ZYM, a semiquantitative colourimetric kit (Dani et al. 2005).

Our aim is to describe components of parasitoid wasp venom and better understand its enzymatic diversity. In the present study, the presence of an ALPase in the venom apparatus of an endoparasitoid wasp, *Pteromalus puparum* L. (Hymenoptera: Pteromalidae) is described. This enzyme was partially characterized at the biochemical level, its coding gene cloned and its mRNA expression levels investigated.

## Methods and Materials

### Insect rearing

The colony of *P. puparum* was maintained as described by Cai et al. (2004). Following the eclosion of wasps, female adults were collected in glass vials (50 × 230 mm) and fed with 20% (v/v) honey solution absorbed on cotton at 25 ± 1° C with a photoperiod of 10:14 (L:D).

### Enzyme histochemistry

5-bromo-4-chloro-3′-indolyl phosphate (BCIP) and nitro blue tetrazolium (NBT) (Promega, www.promega.com) staining followed the method of Yang et al. (2000) with some modifications. Briefly, the venom apparatus was soaked in 4% (v/v) paraformaldehyde for 20 min. The venom apparatus was then treated with 0.1% Triton in PBS for 30 min and washed 3 times for 5 min with PBS. Finally, the venom apparatus was labeled using the chromogenic substrate of BCIP/NBT in the dark until the color developed. It was then washed 3 times for 5 min with PBS. The BCIP/NBT labeling reaction contained 0.17 mg/ml BCIP and 0.33 mg/ml NBT in reaction buffer (100 mM Tris-Cl, 100 mM NaCl, 5 mM MgCl_2_, pH 9.5). The control venom apparatus was processed in the same way with the exception that NBT/BCIP was not added. Color development was viewed under a dissecting microscope and photographed with an Auto-Montage camera system.

For electron microscopy, the method was performed as described by Sarathchandra et al. (2005) modified from Rees and Ali (1988). The procedures for treating the samples were as follows: (1) fixed with 2.5% glutaraldehyde in 0.1 M sodium cacodylate buffer (pH 7.4) at 4° C for 1 h; (2) washed in 0.1 M sodium cacodylate buffer for 3 times for 10 min; (3) incubated with 40 mM Tris/HC1 buffer (pH 9.0) containing 9 mM sodium β glycerophosphate, 5 mM magnesium chloride and 3.6 mM lead Nitrate for 4 h at room temperature; (4) washed briefly in 0.1 M sodium cacodylate buffer (pH 7.4); (5) post-fixed in OSO4 in 0.1 M cacodylate buffer for 2 h; and (6) dehydrated in a graded 50–100% acetone series and then embedded in Epon 812. In control samples, β-glycerophosphate was not added to the incubation medium. The ultrathin sections were observed using a JEX-1230 transmission electron microscope (JEOL, Tokyo) at an accelerating voltage of 80 kV.

### Enzyme Assay

The crude venom was prepared in sterile distilled water as described by Zhang et al. (2005). The enzyme activity was determined according to the method of Dani et al. (2005) using *p*-nitrophenyl phosphate (*p*-NPP) as the substrate at 45° C with one modification that the acid buffer was instead by 0.1 M glycine-NaOH alkaline buffer (pH 8.5). The amount of *p*-nitrophenol (*p*-NP) released was measured using a microplate reader (Bio-Tek Instruments) at 405 nm. The thermal denaturalization of enzyme was analyzed at 40, 50 and 60° C with incubation times of 5, 10, 20 and 30 min. To study the effects of metals (Mg^2+^, Ca^2+^, Zn^2+^, Mn^2+^), the final concentrations ranged from 0 to 5 mM. To determine the time-course of enzyme activity, samples of crude venom from the adults 0 to 7 days after eclosion were assayed, and 100 venom reservoir equivalents (VREs) (one VRE being defined as the supernatant from one torn venom reservoir in 1 µl sterile distilled water) per assay was used. The specific activities for one VRE were calculated using a nitrophenol calibration curve produced using a standard solution of *p*-NPP (Sigma, www.sigmaaldrich.com). The assays not added venom samples were used as controls.

### Molecular cloning of ALPase

Venom apparatus SMART cDNA library (Zhu et al. 2008) was used as the template in all subsequent PCR amplifications. Degenerate primers, forward primer (5′-GTNGARGGNG GNAARATHGA-3′) and reverse primer (5′TCYTCNCCNCCRTGNGTYTC-3′) were designed based on highly conserved amino acid sequences VEGGKID and ETHGGED found in several insects. They were used to amplify a cDNA fragment in a PCR reaction with an initial denaturation step at 94° C for 3 min, 40 cycles running each with 94° C for 30 s, 55° C for 30 s, and 72° C for 1 min, and 10 min at 72° C for extension. For full length cDNA cloning, the RACE protocol was performed according to the instructions of the SMART™ RACE cDNA amplification kit (Clontech, www.clontech.com). Gene specific primers, (5′-CGACGAGAACAGGAAGGACCCGTTTTAT-3′) and (5′-GTTGCCTCGTTCTGGGTAGCCGTTCAT-3′) were designed to amplify the 3′- and 5′-ends of the cDNA fragment, respectively in combination with CDS III/3′ PCR primer and SMART IV oligonucleotide primer.

The PCR products were gel extracted (QIAquick-Gel Extraction Kit, www.qiagen.com) and sequenced according to the dideoxy method with CEQ Dye Terminator using an ABI Prism 377 DNA sequencer (Applied Biosystems, www.appliedbiosystems.com). Sequence similarity was assessed using BLASTP against the NCBI database (Altschul et al. 1997). Multiple alignments were performed with CLUSTAL X, version 1.83 (Thompson et al. 1997). Signal peptide predictions were made using the SignalP (Bendtsen et al. 2004), and motifs were searched with Motifscan (Falquet et al. 2002).

### Semiquantitative RT-PCR

Venom apparatus from 0 to 7 days old *P. puparum* females were dissected and their RNA samples were extracted using Trizol reagent™ (Invitrogen, www.invitrogen.com). Total RNA samples were quantified to the same content and were treated with RQI RNase-free DNase (Promega, www.promega.com) to eliminate possible trace amounts of DNA contamination according to the manufacturer's instructions. The cDNA templates were synthesized using a RevertAid ™ First Strand cDNA Synthesis Kit (MBI, Fermentas, www.fermentas.com). To control for genomic DNA contamination, the total RNA samples that were not reverse transcribed into cDNA were used as the control templates. Two gene-specific primers were (forward, 5′-CATCACCAGAACCACGCAC-3′ and reverse, 5′- TCTTTGAAGCAAGCCGCAT-3′). PCRs were standardized using 18S rRNA specific primers (forward, 5′CGAGCGATGAACCGACAGC-3′ and reverse, 5′-ATTGGAGGGCAAGTCTGGTG-3′). PCR cycling conditions were as follows: 94° C (3 min) followed by 40 cycles (30 cycles for 18s rRNA) at 94° C (30 s), 52° C (30 s) (50° C for 18s rRNA), and 72° C (1 min), and then 1 cycle of 72° C for 10 min. PCR products were then visualized with ethidium bromide on 1% agarose gels.

**Figure 1:**
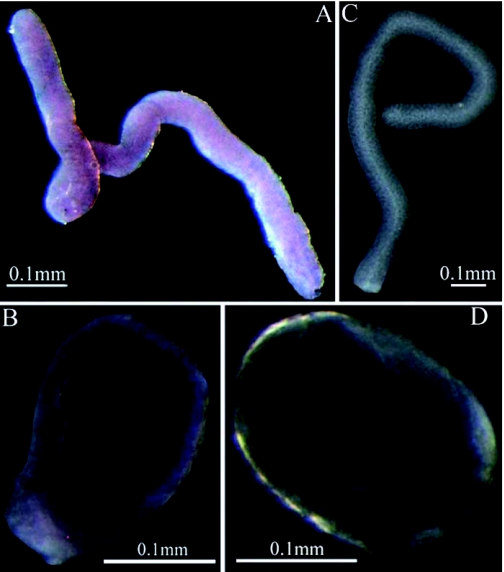
Localization of ALPase activity in the venom apparatus of *Pteromalus puparum* by BCIP/NBT staining. (A) ALPase activity in the venom gland; (B) ALPase activity in the venom reservoir; (C and D) Control venom gland and venom reservoir subjected to ALPase staining protocol without chromogenic substrate. High quality figures are available online.

**Figure 2:**
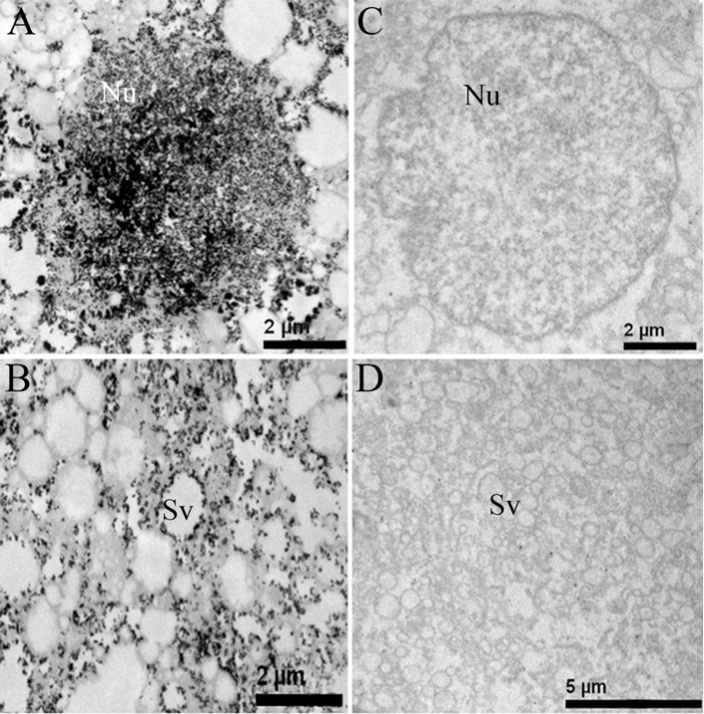
Ultrastructural localization of ALPase in *Pteromalus puparum* venom gland secretory cells. Black lead deposits showing the presence of ALPase. (A–B) Observe the presence of this enzyme inside nuclei (Nu) and secretory vesicles (Sv). (C–D) Negative controls. High quality figures are available online.

## Result

### Histochemical detection of ALPase

Using chromogenic substrates BCIP and NBT, the localization of ALPase activity was examined in the venom apparatus of *P. puparum* by light microscopy (Figure 1). Within the venom gland, ALPase activity was detected in whole mount of this tissue, while color development was weak (Figure 1A). In contrast, the venom reservoir stained strongly positive for ALPase activity, indicating the high amount of ALPase contained in this tissue (Figure 1B). In addition to light microscopy, the presence of ALPase in the venom apparatus was also detected as depicted in Figure 2 using the *p*-NPP technique for histochemical localization by electron microscopy. The black deposit of lead phosphate indicative of phosphatase activity was observed along the inner margin of the secretory vesicles and within the nuclei (Figures 2A and B). Controls showed no positive reaction, as no black deposit was produced (Figures 2C and D).

**Figure 3:**
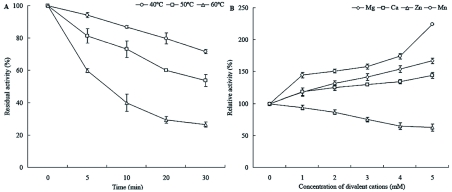
Biochemical characterization of venom ALPase of *Pteromalus puparum*. (A) Thermal stability of venom ALPase of *P. puparum*. The residual activity of the enzyme was determined at pH 8.5 following heating for 0–30 min. (B) Effect of divalent cations (Mg, Ca, Zn and Mn) on ALPase activity in the venom apparatus of *P. puparum*. Values are the mean of three independent assays, and error bars are ± SD of the mean. High quality figures are available online.

### Thermal stability of ALPase

In order to determine a possible modulation of the ALPase activity by thermal denaturalization, the activity was measured by preincubation of the crude venom at 40, 50 and 60° C for 5, 10, 20 and 30 min and then *p*-NPP was added as substrate to start the reaction. Figure 3A shows the time course of the ALPase thermal stability. There was only a very slight decrease in *p*-NPP hydrolysis with time after pre-incubation at 40° C, but higher temperatures markedly inactivated the enzyme. The ALPase retained 71.88% of its initial activity after pre-incubation for 30 min at 40° C, 53.87% activity at 50° C and 26.70% at 60° C.

### Effect of divalent cations on ALPase activity

The effects of different divalent cations at different concentrations of ALPase activity were studied (Figure 3B). Mg^2+^, Ca^2+^ and Mn^2+^ affected the activity of ALPase but to differing extents. At concentrations of 0 to 5 mM, ALPase activity was steadily activated by these three cations. With 5 mM Mg^2+^ and Ca^2+^, the enzyme activity increased to 164.18% and 144.41% compared with the control, respectively. In contrast to Mg^2+^ and Ca^2+^, the effect of Mn^2+^ on the activity of ALPase was higher with an increase to 224.36%. However, the presence of Zn^2+^ resulted in inactivation of the enzyme activity. The activity of ALPase markedly decreased with increasing Zn^2+^ concentrations. The activity was reduced to 62.75% with 5 mM Zn^2+^.

### cDNA clone of ALPase

**Figure 4:**
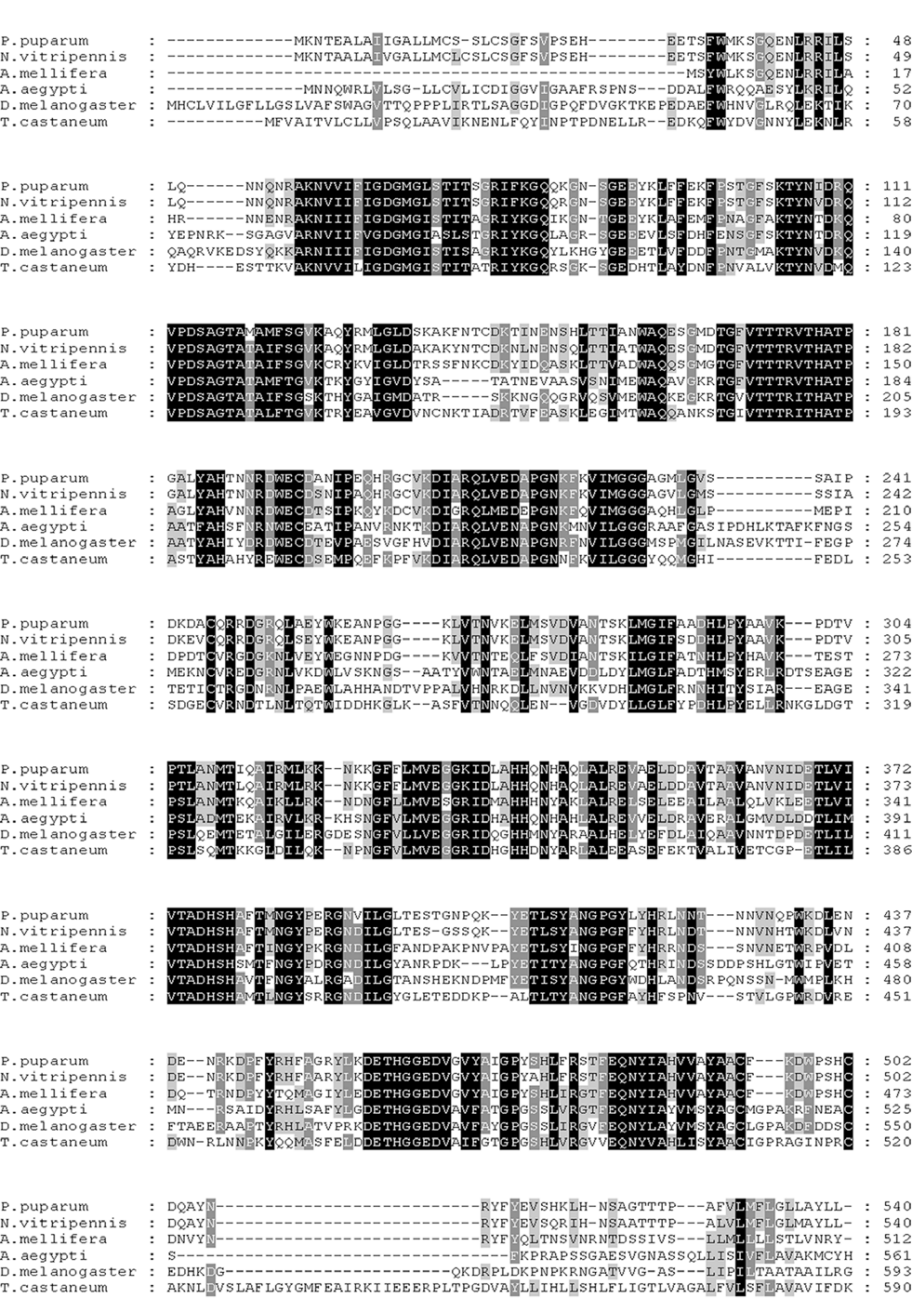
Multiple alignment of the deduced amino acid of venom ALPase amino acid sequence from *Pteromalus puparum* with the counterparts from other species. The sequences used in the alignment are *Nasonia vitripennis* (accession number XP_001603241 ), *Apis mellifera* (accession number XP_624078), *Tribolium castaneum* (accession number XP_9750S0), *Aedes aegypti* (accession number XP_001657478) and *Drosophila melanogaster* (accession number NP_524601). Identical amino acids are shaded in black, and similar amino acids are in grey. High quality figures are available online.

The amplification of venom ALPase of *P. puparum* using two degenerate primers designed based on the conserved amino acid sequences yielded the expected PCR product of approximately 400 bp in length. The sequence of the cloned fragment showed significant similarity to reported ALPases available in the NCBI database. In order to obtain the full-length sequence, two primers were then designed as the gene-specific primers from the known fragment for 3′ and 5′ RACE. PCR of 3′ and 5′ RACE resulted in amplicons of 721 and 1787 bp, respectively. After assembling these three fragments, the full length ALPase was a total of 2645 bp long (Accession number: EU726269). The open reading frame (ORF) was 1623 bp long beginning with the methionine start codon ATG and ending with translation stop codon TAG at nucleotide positions 615 and 2,237, respectively. The cDNA contained a 5′ untranslated region (5′-UTR) of 614 bp and a 3′ untranslated region (3′-UTR) of 408 bp, including a poly (A) tail of 28 bp. The predicted molecular mass of the ALPase deduced amino acid sequence was calculated to be 59.83 kDa and the predicted calculated isoelectric point was 6.98. A BLASTP (Altschul et al., 1997) search revealed that the deduced protein sequence had strong homology with ALPase of the other insect species (*N. vitripennis* 91%, *Apis mellifera* 66%, *Tribolium castaneum* 48%, *Anopheles gambiae* 48%, *D. melanogaster* 46%). The alignment of the venom ALPase of *P. puparum* with that of these insects is reported in Figure 4. Analysis of the deduced amino acid sequence by Signal P (Bendtsen et al. 2004) showed the presence of a signal peptide of 22 predominantly hydrophobic amino acids (MKNTEALAIIGALLMCSSLCSG) at its N-terminus. Motifscan (Falquet et al. 2002) was used to find profiles of the ALPase protein. There was a long, highly conserved ALPase gene family signature at amino acid positions 56–495. Moreover, there were three potential N-glycosylation sites, seven potential casein kinase II phosphorylation sites, nine potential N-myristoylation sites, four potential protein kinase C phosphorylation sites, one potential tyrosine kinase phosphorylation site and several metal ligand sites.

### Time-course related expression

**Figure 5:**
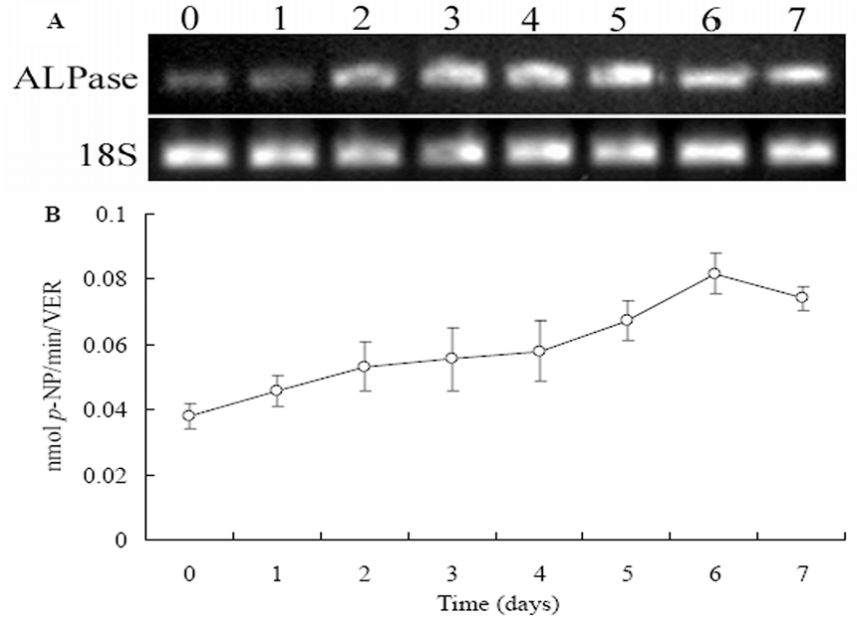
Time-course expression profiles of ALPase in the venom apparatus of *Pteromalus puparum* from 0 to 7 days after emergence. (A) Agarose gels of RT-PCR amplicons for ALPase and 18S at various times post emergence from one of the three experiments. (B) Enzyme activity profiles of ALPase in the venom apparatus at various times post emergence. Values are the mean of three independent assays, and error bars are ± SD of the mean. High quality figures are available online.

Semiquantitative RT-PCR analysis was employed to compare the levels of ALPase transcripts in venom apparatus of *P. puparum* from 0 to 7 days after adult eclosion (Figure 5A). A low ALPase expression was found at days 0 and 1. Thereafter expression levels increased gradually, and remained high on days 6 to 7. Subsequently, ALPase activity was measured from 0 day post female adult eclosion and continued until 7 days (Figure 5B). A clear time related modulation of enzymatic activity in the venom apparatus of *P. puparum* was evident in different days. ALPase activity was observed to be increasing at 0 to 6 days. The maximum peak was seen at day 6. Then, the level showed a slight decrease at day 7. The results revealed that the time-dependent changes in the gene expression of ALPase mRNA that corresponds to the alterations in ALPase enzymatic activity.

## Discussion

In the present study, the histochemical results demonstrated that ALPase is present in the venom apparatus of *P. puparum*. From the *P. puparum* venom apparatus ultrastructural study (Zhu et al. 2007), we know that the main function of the venom gland is to produce the venom that is stored in the venom reservoir. According to the BICP/NBT staining, a slight color development in the venom gland was observed, while the venom reservoir was deeply stained suggesting that ALPase was produced in the venom gland and then accumulated in the venom reservoir. ALPase activity was located in the secretory vesicles and nuclei of venom gland using *p*-NPP technique and electron microscopy.

ALPase has been shown to be located on the cell surface, linked to the cell membrane via a phosphatidylinositol glycan linkage (Low and Saltiel 1988). This is not the case in the venom gland secretory cells of *P. puparum* as ALPase was located in all parts of the nuclei and in periphery of the secretory vesicles. Therefore, we suggest that *P. puparum* venom ALPase is an intracellular enzyme.

ALPase has multifunctional roles in cell biology. In bacterial cells, this enzyme was reported to be located in the periplasmic space as a homodimer involved in the acquisition of phosphate from esters (Suzuki et al. 2005). In human cells, ALPase is important for regulating B cell differentiation and maturation (Hossain and Jung 2008). In rat intestinal epithelial cells, ALPase activity is associated with morphological alterations (Wood et al. 2003). In insects, acid phosphatase activity is commonly related to cellular pathways leading to cell secretion (Eguchi 1995). Although the role of ALPase located in the organelles of *P. puparum* venom gland secretory cells remains to be established, we believe that it could be involved in the venom secretion cycle based on its presence in nuclei and secretory granules that are indicative of protein synthesis and secretion.

It is known that the response of ALPase to heat treatment can vary widely. In *P. puparum*, the thermal denaturalization results indicated that this venom enzyme was weakly resistant to heat treatment, with a 30 min exposure to 60°C resulting in 73.3% inhibition. This observation is consistent with the other reports of the thermal stability of a number of ALPases from mammalian intestine and insect salivary gland which were resistant to heat treatment (Goldstein et al. 1980; Funk 2001). As ALPase is a dimeric metalloenzyme (Eguchi 1995), the effect of different divalent cations on its activity was evaluated. The results showed that venom ALPase activity was reduced by Zn^2+^ agreeing with the observation of Rodrigues et al. (2006) who reported that Zn^2+^ is an inhibitor of ALPase from spider venom. ALPases in a variety of organisms demonstrate activity dependence on Zn^2+^ (Mazorr et al. 2002). A series of studies on insect ALPases showed that it was activated by Mg^2+^ and Ca^2+^ (Terra et al. 1979; Eguchi 1995). Similar results were found for the ALPase from *P. puparum* venom. This enzyme was also highly activated by Mn^2+^. The above results suggest that *P. puparum* venom ALPase is temperature sensitive and sensitive to some metal ions that may play important roles in the enzyme activity. Since only tiny amount of venom is available for recovering from the parasitoid wasps, purification of venom ALPase from whole body homogenates is required. This partial biochemical information is useful for providing some basis to isolate this enzyme.

The venom ALPase gene was cloned from *P. puparum* using a venom apparatus cDNA library as template, providing the first evidence for a clone of this venom proteinase gene from the venom gland of parasitoids. Generally, ALPases in insects are present in different isozymes (Eguchi 1995). In the silkworm, *B. mori*, two ALPase isozymes of mALPase (membrane bound type) and sALPase (soluble type) are known from the larval midgut epithelium (Itoh et al. 1999). The possibility that there are ALPase isozymes present in the venom apparatus of *P. puparum* is raised. It is possible that the ALPase gene cloned here would be just one of the isozymes that are responsible for the enzyme activity detected during the biochemical characterization described above. To solve this problem, efforts to isolate other clones having different nucleotide sequences and southern blot analyses to identify the gene copy number of ALPase in the venom apparatus of *P. puparum* are needed. With regard to the clone isolated in this study, its calculated molecular weight of 59.83 kDa and pi of 6.98 is similar to values previously reported for other insect ALPases (Itoh et al. 1991, 1999). In all reported parasitoid venom proteins (Moreau and Guillot 2005), signal peptides are located in their sequences. *P. puparum* venom ALPase also has this structure.

It is known that signal peptides are functional for directing polypeptides into the endoplasmic reticulum to initiate secretion (Leader 1979). This suggests that venom ALPase of *P. puparum* is derived from the venom apparatus secretory system. This strengthens the above conclusion obtained from histochemical observations that this enzyme is as an intracellular enzyme produced in the venom gland secretory cells and finally secreted into the venom reservoir for storage.

ALPases from insects are post-translationally modified by glycosylation (Itoh et al. 1999; Asgeirsson et al. 2003). In addition to N-glycosylation sites, venom ALPase from *P. puparum* had different numbers of potential phosphorylation and N-myristoylation sites. Concerning the effect of divalent cations on ALPase, residues for metal ligand sites were present in its sequence. Furthermore, sequence alignment analysis demonstrated that the venom protein exhibited features typical of ALPase from other species including the long conserved ALPase gene family signature sequence, suggesting conservation of function.

In living organisms ALPase activity serves a variety of essential functions that include nutrition, phosphate metabolism, intracellular signaling as well as modification and transport of metabolites across biological membranes (Cho-Ngwa et al. 2007). For example, in crabs, ALPase is important in the absorption of phosphate and calcium from seawater and for the shell formation and as potential biochemical indicator of stress (Pinoni et al. 2005). In mammals, there is some experimental evidence that ALPase is involved in tissue development and cellular differentiation, although its precise role in these cellular events is not known (Ali et al. 2005). In bacteria, ALPase plays a key role providing inorganic phosphate from the growth medium (Torriani 1990). It has been suggested that ALPases are involved with nutrient absorption involved in the processes of digestion in insect midgut (Eguchi 1995; Yi and Adams 2001). In keeping with this hypothesis, it is worth suggesting that ALPase in parasitoid venom could assist parasitoid absorption of nutrients from host insects. Moreover, parasitoid venom ALPase could be potentially involved in many other functions as occurs in other organisms. New experiments with these speculations are needed to understand the function of ALPase in parasitoid venom.

In parasitoid wasps, two venom genes, phenoloxidase and acid phosphatase, had specific expression patterns that responded during development (Parkinson et al. 2001; Zhu et al. 2008). It is important to investigate the changes of venom ALPase gene expression after female adult wasp eclosion. During days 0 to 7 after eclosion, RT-PCR analysis showed that gene transcription of venom ALPase increased. By comparison with the mRNA level, the change profiles of enzymatic activities found in the venom apparatus of *P. puparum* were in accord with gene transcription during the time interval observed. Like venom phenoloxidase and acid phosphatase (Parkinson et al. 2001; Zhu et al. 2008), we suggest that transcriptional activity of venom ALPase would be also correlated with the oviposition process.
